# Supplementary material for: Machine learning prediction of motor response after deep brain stimulation in Parkinson’s disease—proof of principle in a retrospective cohort
Source: PeerJ. 2020 Nov 18;8:e10317. doi: 10.7717/peerj.10317 (PMC7680051; doi:10.7717/peerj.10317)
Supplement: Supplemental Information 1 [file peerj-08-10317-s001.docx]

**Supplementary material of manuscript ‘Machine learning prediction of motor response after deep brain stimulation in Parkinson's disease – proof of principle in a retrospective cohort’ by Habets et al.**

**Legend**

1. Surgical procedure
2. The applied categorization for postoperative motor response
3. Literature on clinically relevant UPDRS differences
4. Imputation methods
5. References
6. **Surgical procedure**

PD patients were indicated for STN DBS based on severe motor symptoms despite optimal levodopa treatment, severe motor fluctuations, or dyskinesia, and often showed a good levodopa responsiveness.(Temel et al. 2007) Surgical electrode location was determined based on preoperative MRI trajectory planning, microelectrode recordings, and intra-operative testing. The first part of surgery was performed while the patient was cognizant, after which the lead and pacemaker implantation was completed under general anaesthesia. Postoperative CT examinations verified the electrode location. Postoperative stimulation parameters and dopaminergic drug therapy were set and managed by the neurologist in the outpatient clinic during regular follow-ups.

1. **The applied categorization for postoperative motor response**

Quantifying motor response after STN DBS is a challenging task at its own due to the variety in symptoms and the variety in decisive reasons for surgical inclusion. Symptom severity in on-medication state, time spend in off-time, frequency of on- vs. off-fluctuations, dopaminergic adverse effects all could be reasons to include a PD patient for STN DBS. Obviously, successful motor response after STN DBS is as diverse as these different reasons for surgery. Previous literature describes STN DBS outcome with a heterogenous variety of variables including UPDRS III severity in on- or off-conditions, time spent in on-condition, and QoL scales.(Deuschl et al. 2006; Odekerken et al. 2013; Schuepbach et al. 2019; Williams et al. 2010)

To identify the suboptimal responding minority of STN DBS patients objectively, we pragmatically created a holistic categorization based on available variables representing ADL, motor symptoms, and adverse effects (paper fig. 2). The applied cut off values are based on existing literature and are argued in the next paragraph of these supplementary material. In the absence of a validated QoL scale, we consider our categorization as the best possible definition of general unsatisfactory outcome.

This categorization resulted in one-third weak responders, which is comparable to reported improvement ratios on quality of life after STN DBS.(Dafsari et al. 2018; Daniels et al. 2011; Liu et al. 2018; Williams et al. 2010) Especially since we regarded postoperative differences in on-medication conditions instead of off-medication conditions; and postoperative surgical results are often comparable to the best state in on-medication condition.(Kleiner-Fisman et al. 2006)

The strong and weak responders significantly differed on all UPDRS changes, except for UPDRS III scores in on-stimulation and off-medication condition compared to preoperative off-medication condition (table 1). A plausible explanation is that nearly all patients will benefit from stimulation compared to the untreated preoperative condition. We consider comparing on-medication states as more natural and realistic because therapy aims to keep patients in on-medication state most of the time.

Given the concordance regarding preoperative and postoperative UPDRS scores and LEDD amounts between our cohort and the literature, we consider our cohort to be representative for the general STN DBS population.(Deuschl et al. 2006; Williams et al. 2010) Our cohort showed an expected older age and more severe pre- and postoperative characteristics then a cohort included based on early motor complications.(Schuepbach et al. 2013) The observed neuropsychological deteriorations after STN DBS are also in line with previous findings.(Okun 2012)

1. **Literature on clinically relevant UPDRS differences**

| **Reference** | **Compared (MDS-) UPDRS score** | **Sign difference** |
| --- | --- | --- |
| Kostoglu(Kostoglou et al. 2017) | III Med-OFF vs. Med-OFF + STIM | 38% |
| Horvath(Horvath et al. 2015) | III | - 3.25  + 4.63 |
| Shulman(Shulman et al. 2010) | III  Total | +/- 2.5 (minimal)  +/- 5.2 (moderate)  +/- 10.8 (large)  +/- 4.3 (minimal)  +/- 9.1 (moderate)  +/- 17.1 (large) |
| Schrag(Schrag et al. 2006) | III  II (H&Y > 2) | +/- 5  3 |
| Makkos(Makkos et al. 2018) | II + III  I + II + III  Total | -4.9  + 4.2  -6.7  +5.2  -7.1  +6.3 |

**Table 1: overview clinically significant changes in UPDRS scores reported in literature.**

We searched PubMed using search terms: (Parkinson*) AND (UPDRS) AND ((clinical* relevant) OR (clinical* significant)) AND (improve* OR change OR difference). We selected papers which aimed to define minimal clinically relevant UPDRS changes and included predictive papers which used absolute thresholds for UPDRS changes in defining outcome.

Table 1 shows the reported UPDRS differences suggested to be clinically relevant.

For the UPDRS III score, the relevant differences range between 2.5 and 5 for the smallest relevant change. In one predictive analysis, a cut off change of 38% was used for UPDRS III off-medication. This translates to an absolute change of >10 in many patients. We averaged these findings and set the cut off for clinically relevant improvement on UPDRS III at 5 points.

Since the UPDRS II and IV scores consist of fewer points, it is logical that the cut off values are lower. Based on the mentioned cut offs in the literature, we set the cut off for the UPDRS II and IV scores at 3 points. Every patient who improved more than 3 points on UPDRS II or IV was evaluated on UPDRS III change. When a patient deteriorated more than 7 points on UPDRS III, while improving on UPDRS II or IV, he or she was classified as a Weak responder. The 7 points cut off on UPDRS III represents the minimal clinical important difference of 5 points, and the natural disease progression of 2 points on UPDRS III scale per year(Holden et al. 2018).

1. **Imputation methods**

We imputed missing values in the presurgical parameters using Random Forrest Regressors or Classifiers for each individual parameter. The models were trained on all participants that had the data available while missing values were imputed based on the available presurgical parameters for the remaining participants. For continuous parameters, we used Random Forrest Regressors while categorical parameters were imputed using Random Forrest Classifiers. Only presurgical parameters were imputed to ensure that our results were not based on imputed values.

1. **References**

Dafsari HS, Weiss L, Silverdale M, Rizos A, Reddy P, Ashkan K, Evans J, Reker P, Petry-Schmelzer JN, Samuel M, Visser-Vandewalle V, Antonini A, Martinez-Martin P, Ray-Chaudhuri K, Timmermann L, Europar, and the INMPDSG. 2018. Short-term quality of life after subthalamic stimulation depends on non-motor symptoms in Parkinson's disease. *Brain Stimul*. 10.1016/j.brs.2018.02.015

Daniels C, Krack P, Volkmann J, Raethjen J, Pinsker MO, Kloss M, Tronnier V, Schnitzler A, Wojtecki L, Botzel K, Danek A, Hilker R, Sturm V, Kupsch A, Karner E, Deuschl G, and Witt K. 2011. Is improvement in the quality of life after subthalamic nucleus stimulation in Parkinson's disease predictable? *Mov Disord* 26:2516-2521. 10.1002/mds.23907

Deuschl G, Schade-Brittinger C, Krack P, Volkmann J, Schafer H, Botzel K, Daniels C, Deutschlander A, Dillmann U, Eisner W, Gruber D, Hamel W, Herzog J, Hilker R, Klebe S, Kloss M, Koy J, Krause M, Kupsch A, Lorenz D, Lorenzl S, Mehdorn HM, Moringlane JR, Oertel W, Pinsker MO, Reichmann H, Reuss A, Schneider GH, Schnitzler A, Steude U, Sturm V, Timmermann L, Tronnier V, Trottenberg T, Wojtecki L, Wolf E, Poewe W, and Voges J. 2006. A randomized trial of deep-brain stimulation for Parkinson's disease. *N Engl J Med* 355:896-908. 10.1056/NEJMoa060281

Holden SK, Finseth T, Sillau SH, and Berman BD. 2018. Progression of MDS-UPDRS Scores Over Five Years in De Novo Parkinson Disease from the Parkinson's Progression Markers Initiative Cohort. *Mov Disord Clin Pract* 5:47-53. 10.1002/mdc3.12553

Horvath K, Aschermann Z, Acs P, Deli G, Janszky J, Komoly S, Balazs E, Takacs K, Karadi K, and Kovacs N. 2015. Minimal clinically important difference on the Motor Examination part of MDS-UPDRS. *Parkinsonism Relat Disord* 21:1421-1426. 10.1016/j.parkreldis.2015.10.006

Kleiner-Fisman G, Herzog J, Fisman DN, Tamma F, Lyons KE, Pahwa R, Lang AE, and Deuschl G. 2006. Subthalamic nucleus deep brain stimulation: summary and meta-analysis of outcomes. *Mov Disord* 21 Suppl 14:S290-304. 10.1002/mds.20962

Kostoglou K, Michmizos KP, Stathis P, Sakas D, Nikita KS, and Mitsis GD. 2017. Classification and Prediction of Clinical Improvement in Deep Brain Stimulation From Intraoperative Microelectrode Recordings. *IEEE Trans Biomed Eng* 64:1123-1130. 10.1109/tbme.2016.2591827

Liu FT, Lang LQ, Yang YJ, Zhao J, Feng R, Hu J, Wang J, and Wu JJ. 2018. Predictors to quality of life improvements after subthalamic stimulation in Parkinson's disease. *Acta Neurol Scand*. 10.1111/ane.13056

Makkos A, Kovacs M, Aschermann Z, Harmat M, Janszky J, Karadi K, and Kovacs N. 2018. Are the MDS-UPDRS-Based Composite Scores Clinically Applicable? *Mov Disord* 33:835-839. 10.1002/mds.27303

Odekerken VJ, van Laar T, Staal MJ, Mosch A, Hoffmann CF, Nijssen PC, Beute GN, van Vugt JP, Lenders MW, Contarino MF, Mink MS, Bour LJ, van den Munckhof P, Schmand BA, de Haan RJ, Schuurman PR, and de Bie RM. 2013. Subthalamic nucleus versus globus pallidus bilateral deep brain stimulation for advanced Parkinson's disease (NSTAPS study): a randomised controlled trial. *Lancet Neurol* 12:37-44. 10.1016/s1474-4422(12)70264-8

Okun MS. 2012. Deep-brain stimulation for Parkinson's disease. *N Engl J Med* 367:1529-1538. 10.1056/NEJMct1208070

Schrag A, Sampaio C, Counsell N, and Poewe W. 2006. Minimal clinically important change on the unified Parkinson's disease rating scale. *Mov Disord* 21:1200-1207. 10.1002/mds.20914

Schuepbach WM, Rau J, Knudsen K, Volkmann J, Krack P, Timmermann L, Halbig TD, Hesekamp H, Navarro SM, Meier N, Falk D, Mehdorn M, Paschen S, Maarouf M, Barbe MT, Fink GR, Kupsch A, Gruber D, Schneider GH, Seigneuret E, Kistner A, Chaynes P, Ory-Magne F, Brefel Courbon C, Vesper J, Schnitzler A, Wojtecki L, Houeto JL, Bataille B, Maltete D, Damier P, Raoul S, Sixel-Doering F, Hellwig D, Gharabaghi A, Kruger R, Pinsker MO, Amtage F, Regis JM, Witjas T, Thobois S, Mertens P, Kloss M, Hartmann A, Oertel WH, Post B, Speelman H, Agid Y, Schade-Brittinger C, and Deuschl G. 2013. Neurostimulation for Parkinson's disease with early motor complications. *N Engl J Med* 368:610-622. 10.1056/NEJMoa1205158

Schuepbach WMM, Tonder L, Schnitzler A, Krack P, Rau J, Hartmann A, Halbig T, Pineau F, Falk A, Paschen L, Paschen S, Volkmann J, Dafsari HS, Barbe MT, Fink GR, Kuhn A, Kupsch A, Schneider GH, Seigneuret E, Fraix V, Kistner A, Chaynes PP, Ory-Magne F, Brefel-Courbon C, Vesper J, Wojtecki L, Derrey S, Maltete D, Damier P, Derkinderen P, Sixel-Doring F, Trenkwalder C, Gharabaghi A, Wachter T, Weiss D, Pinsker MO, Regis JM, Witjas T, Thobois S, Mertens P, Knudsen K, Schade-Brittinger C, Houeto JL, Agid Y, Vidailhet M, Timmermann L, and Deuschl G. 2019. Quality of life predicts outcome of deep brain stimulation in early Parkinson disease. *Neurology*. 10.1212/wnl.0000000000007037

Shulman LM, Gruber-Baldini AL, Anderson KE, Fishman PS, Reich SG, and Weiner WJ. 2010. The clinically important difference on the unified Parkinson's disease rating scale. *Arch Neurol* 67:64-70. 10.1001/archneurol.2009.295

Temel Y, Wilbrink P, Duits A, Boon P, Tromp S, Ackermans L, van Kranen-Mastenbroek V, Weber W, and Visser-Vandewalle V. 2007. Single electrode and multiple electrode guided electrical stimulation of the subthalamic nucleus in advanced Parkinson's disease. *Neurosurgery* 61:346-355; discussion 355-347. 10.1227/01.neu.0000303993.82149.98

Williams A, Gill S, Varma T, Jenkinson C, Quinn N, Mitchell R, Scott R, Ives N, Rick C, Daniels J, Patel S, and Wheatley K. 2010. Deep brain stimulation plus best medical therapy versus best medical therapy alone for advanced Parkinson's disease (PD SURG trial): a randomised, open-label trial. *Lancet Neurol* 9:581-591. 10.1016/s1474-4422(10)70093-4
